# Neurovascular coupling is altered in women who have a history of brain injury from intimate partner violence: a preliminary study

**DOI:** 10.3389/fgwh.2024.1344880

**Published:** 2024-03-01

**Authors:** Colin Wallace, Jonathan D. Smirl, Shambhu P. Adhikari, K. Elisabeth Jones, Matt Rieger, Krystal Rothlander, Paul van Donkelaar

**Affiliations:** ^1^ School of Health and Exercise Sciences, University of British Columbia, Kelowna, BC, Canada; ^2^ Department of Kinesiology, Okanagan College, Penticton, BC, Canada; ^3^ Sport Injury Prevention Research Centre, Faculty of Kinesiology, University of Calgary, Calgary, AB, Canada; ^4^ Cerebrovascular Concussion Laboratory, Faculty of Kinesiology, University of Calgary, Calgary, AB, Canada; ^5^ Hotchkiss Brain Institute, University of Calgary, Calgary, AB, Canada; ^6^ Integrated Concussion Research Program, University of Calgary, Calgary, AB, Canada; ^7^ Alberta Children’s Hospital Research Institute, University of Calgary, Calgary, AB, Canada; ^8^ Libin Cardiovascular Institute of Alberta, University of Calgary, Calgary, AB, Canada; ^9^ Faculty of Medicine, University of Alberta, Edmonton, AB, Canada

**Keywords:** intimate partner violence, brain injury, strangulation, neurovascular coupling, cerebrovascular physiology, mental health

## Abstract

**Introduction:**

Intimate partner violence (IPV) is a global health crisis with 30% of women over the age of 15 experiencing at least one event in their lifetime. Brain injury (BI) due to head impacts and/or strangulation is a common but understudied part of this experience. Previous research has shown BI from other injury mechanisms can disrupt neurovascular coupling (NVC). To gain further insight into whether similar changes occur in this population, we assessed NVC responses in women with a history of IPV-BI.

**Methods:**

NVC responses were measured for the middle and posterior cerebral arteries (MCA, PCA) using transcranial Doppler ultrasound while participants performed a complex visual search task. The lifetime history of previous exposure to IPV-BI was captured using the Brain Injury Severity Assessment (BISA) along with measures of post-traumatic stress disorder (PTSD), anxiety, depression, substance use, and demographic information. Initial analyses of NVC metrics were completed comparing participants who scored low vs. high on the BISA or did or did not experience non-fatal strangulation followed by a stepwise multiple regression to examine the impact of PTSD, anxiety, and depression on the relationship between the NVC metrics and IPV-BI.

**Results:**

Baseline and peak cerebral blood velocity were higher and the percentage increase was lower in the PCA in the low compared to the high BISA group whereas no differences between the groups were apparent in the MCA. In addition, those participants who had been strangled had a lower initial slope and area under the curve in the PCA than those who had not experienced strangulation. Finally, the stepwise multiple regression demonstrated the percentage increase in the PCA was significantly related to the BISA score and both depression and anxiety significantly contributed to different components of the NVC response.

**Conclusions:**

This preliminary study demonstrated that a lifetime history of IPV-BI leads to subtle but significant disruptions to NVC responses which are modulated by comorbid depression and anxiety. Future studies should examine cerebrovascular function at the acute and subacute stages after IPV episodes to shed additional light on this experience and its outcomes.

## Introduction

Intimate partner violence (IPV) is an international public health crisis as, on average, 30% of women over the age of 15 report an incident of IPV in their lifetime (1–3). The prevalence of IPV varies extensively based on geographical region, with some areas of the world reporting rates as high as 66% (2). A systematic review by Stöckl *et al*. (2013) examining intimate partner homicide reported one in seven homicides are committed by an intimate partner and the proportion of women homicide victims killed by an intimate partner is six times higher than it is for men (4). In 2009, 335,697 Canadians experienced a total of 942,000 violent encounters at the hands of their intimate partner; the associated economic impact of this violence is estimated at $7.4 billion per year (5), with the COVID-19 pandemic unfortunately exacerbating this issue (6). Women who have experienced IPV commonly report injuries to the head, face and neck sustained during a violent event—Jackson *et al*. (2002) found 92% of women recruited from women's shelters or emergency rooms were hit in the head or face by their partner (7). Thus it stands to reason that a significant portion of this population are at risk of suffering a brain injury (BI) (8). Further, as many as 76% of women who have experienced an IPV event report experiencing non-fatal strangulation (NFS), a method of violence that can lead to unconsciousness within seconds and brain death within minutes and, for women surviving an IPV event, chronic symptoms including, but not limited to, depression, anxiety and posttraumatic stress disorder (PTSD) (9–11). Researchers have examined the incidence of IPV-BI and found it is especially prevalent in this population, as 35%–88% of women who have experienced IPV are diagnosed with a BI, and more than half of women who experience IPV-related injuries suffer multiple partner-related BIs (12–17). To put the endemic nature of this into perspective, this means up to 296,000 women suffer a BI at the hands of an intimate partner each year in Canada. This number does not reflect the true weight of this issue, as most women do not seek medical attention for abuse-related injuries (12).

Little is known about both the short- and long-term consequences of IPV-BI from both a physiological and psychological perspective. A BI results in tissue deformation and direct damage to vascular, neuronal and glial structures due to biomechanical forces (18). As a result, IPV survivors are subject to both acute and chronic mental and physical health consequences including PTSD, increased suicidality, anxiety, depression, decreased cognitive function, substance use and physical injury (12, 19–23). Despite the high incidence of IPV-BI, there has been relatively little quantitative analysis on the associations between the severity and frequency of BI and the resulting pathophysiological and psychopathological consequences. Indeed, the sequelae resulting from a single or multiple IPV-BI events may be compounded by the fact most women do not report their injuries for fear of retribution or because the woman does not recognize the BI (24).

Accurate identification of the presence and severity of BI is contingent on early screening and requires a multidisciplinary approach; however, current clinical exams used in the detection of BI rely heavily on reported symptoms during an interview (18). Missed diagnosis of BI may lead to the exacerbation of symptoms, prolonged neurological and physiological impairments, and a lengthened recovery period. The Brain Injury Severity Assessment (BISA) tool was created in 2003 and involves a semi-structured interview that identifies the history of non-partner and partner-related BI (12). It results in a score from 0 to 8 where lower scores (i.e., 0–4) represent little to no previous exposure to potential BIs resulting from episodes of IPV and higher scores (i.e., 5–8) represent significant exposure to IPV-BI with episodes happening more frequently, more recently, and with more severe consequences including potential loss of consciousness and post-traumatic amnesia. The BISA has been shown to be related to disruptions to neurocognitive function, white matter integrity, and functional connectivity (12, 25).

Finally, BIs experienced by women surviving IPV are associated with an increased risk of developing Alzheimer's disease (AD) and may have the potential to lead to chronic traumatic encephalopathy (CTE) analogous to that experienced by many former collision sport athletes (26, 27). Thus, a better understanding of the physiology surrounding IPV-BI is of vital importance to improving long-term health outcomes in this underserved population. It has been suggested alterations in cerebrovascular regulation induced by BI can lead to an increased risk of developing the cognitive impairment underlying dementia and AD later in life (28, 29). Hence, in the current study we examined cerebrovascular regulation in women who have experienced IPV-BI.

Transcranial Doppler ultrasound (TCD) has shown remarkable value in the assessment of multiple domains of cerebrovascular function, including elevations in cerebral blood velocity (CBv) due to increases in cerebral neuronal metabolism (activation of neural tissue), referred to as neurovascular coupling (NVC) (30, 31). In particular, NVC reflects the maintenance of appropriate levels of nutrient and oxygen supply to relevant brain regions during functional activation (30). Alterations to cerebral blood flow regulatory mechanisms are associated with hypertension and AD, and it has been postulated that NVC is useful in quantifying the degree of vascular and metabolic decoupling in these conditions (32–34). We have previously examined NVC metrics following acute sport-related concussion (SRC) and found altered CBv responses in athletes up to two weeks following injury compared to non-injured controls (35). Determining whether cerebral blood flow changes are the cause or consequence of alterations in functional activation following BI is challenging. Preclincial animal models offer perhaps the clearest insight into this issue and have shown both immediate effects of head impacts on neurovascular structure and function most likely due to cerebral vascular injury/disruption as well as mid- and long-term alterations in cerebral blood flow and brain activation that are interrelated and, therefore, difficult to disentangle [reviewed in (30)].

Previous work has shown that the cumulative effects of BI may make an individual more susceptible to subsequent injury, leading to lingering symptoms and a lengthier recovery period (36). As most women who have experienced IPV endure numerous events before seeking shelter, it is important to identify the presence and severity of symptoms and brain dysfunction to direct appropriate treatment and rehabilitation strategies. Assessment of NVC through TCD may provide further evidence for the challenges these individuals are working to overcome on a daily basis. No previous study has investigated the extent to which NVC metrics are altered in the context of IPV-BI, the influence on BI severity and occurrence of NFS on the magnitude of NVC alterations, and the prospective role of TCD in identifying the history, presence and severity of BI in this population. Thus, the objective of this study were to evaluate the effects of IPV-BI on NVC dynamics. It was hypothesized (i) NVC responses would be disrupted in women who have experienced IPV-BI, (ii) NFS would affect NVC responses above and beyond that due to head impacts alone; and (iii) comorbid factors (PTSD, depression, anxiety) would modulate these effects.

## Materials and methods

### Participants

The principal criterion for study inclusion was at least one reported incident of IPV. Participants were not excluded if they had experienced any form of BI outside the context of IPV. As such, 37 women were recruited from local community partner sites (demographic information can be found in Table 1). All aspects of the study were described to the women prior to written informed consent being provided, with all questions about the study being explained prior to participation. The study was approved by the Clinical Research Ethics Board at the University of British Columbia.

**Table 1:** Participant demographics and clinical characterisics (*N* = 37).

Characteristics	Mean ± SD or *n* (%)
Age, years	37.42 ± 8.49
Education, years	13.36 ± 2.11
Ethnicity, % (*n*)	
Caucasian	22 (59.45)
Indigenous	10 (27.03)
Other/Did not disclose	5 (13.51)
WEB, total score	47.47 ± 13.58
BISA, total score	3.70 ± 2.17
Time since last IPV-BI episode, months	18.39 ± 22.49
Duration of substance use, years	15.97 ± 9.84
CAPS, total score	177.67 ± 71.04
BAI, total score	23.45 ± 13.78
BDI, total score	22.91 ± 13.06
History of non-IPV-related BI, % (*n*)	
Yes	23 (62.2)
No	14 (37.8)

SD, standard deviation; BISA, Brain Injury Severity Assesment; WEB, Women's Experiences with Battering Scale; CAPS, Clinician Administered PTSD Scale for DSM-IV; BAI, Beck's Anxiety Inventory; BDI, Beck's Depression Inventory; IPV, Intimate partner violence; BI, brain injury.

All participants were tested over 2 sessions separated by 3–7 days. Psychopathological assessments and a brief demographic questionnaire were completed during the first session by the research coordinators (KEJ, KR) who are trained clinical social workers with experience working with women who have survived IPV. The assessments included indices of PTSD [Clinician-Administered PTSD Scale (CAPS)] (37), depression [Beck's Depression Inventory (BDI)] (38), anxiety [Beck's Anxiety Inventory (BAI)] (39), history of substance use [Initial Substance Use Scale (40)], history of previous abuse (Women's Experiences with Battering scale (WEB) (41), and the BISA. The second session consisted of laboratory assessments of cerebrovascular, sensorimotor, neurocognitive, and blood biomarker measures. The current paper focuses on the NVC aspect of cerebrovascular function. All women were familiarized to the testing procedures prior to participation and did not exercise or consume caffeine/alcohol 12 h prior to testing (42). Current symptom burden and other data were reported on a subset of this sample in a previous publication (17).

### Transcranial Doppler ultrasound

The posterior cerebral artery (PCA) and middle cerebral artery (MCA) were insonated using two 2-MHz TCD probes (ST3; Spencer Technologies, Seattle, WA, USA) through the temporal acoustic window on the side of the head to record PCA and MCA velocity. The P-1 PCA and M-1 MCA segments were identified and the signals optimized corresponding to the depth of each vessel, velocity of blood flow and waveform produced (31, 43). Participants were fitted with a three-lead electrocardiogram (ECG). Blood pressure (BP) was measured using finger photoplethysmography with a brachial cuff to adjust for height differences between the finger and brachial artery (Finometer PRO; Finapres Medical Systems, Amsterdam, Netherlands). End-tidal pressure of carbon dioxide (P_ET_CO_2_) was sampled with a mouthpiece and monitored with an inline gas analyzer (ML206; ADInstruments, Colorado Springs, CO, USA), calibrated with a known gas concentration prior to each collection. All data were time-aligned and collected at a sampling frequency of 1,000 Hz via an 8-channel PowerLab (ADInstruments) and stored for offline analysis using commercially available software including LabChart (ADInstruments) and Microsoft Excel (Microsoft Corporation; Redmond, WA, USA).

### Experimental protocol

NVC metrics were quantified using a complex visual search task (Where's Waldo) that has been shown to elicit robust changes in PCA velocity (44, 45). Resting physiological data were recorded while sitting quietly for at least 4 min, while baseline CBv was quantified during a 2-min eyes open and a 2-min eyes closed period. Participants were seated in front of the screen (27” Apple iMac, Apple, Cupertino, California, USA) and completed 6–8 cycles of 20 s with their eyes closed followed by 40 s with their eyes open performing the visual search (46). Screen settings were consistent across all subjects. None of the screen parameters were altered between trials or between participants.

### Data processing

Data processing was completed in the same manner as in previous studies from our group examining NVC coupling (44). Briefly, mean BP and CBv traces were calculated following extraction of beat-to-beat heart rate, peak systolic and end diastolic blood pressure, and peak systolic and end diastolic PCA velocity and MCA velocity using the R-R intervals from the electrocardiogram for gating. P_ET_C_O2_ was measured from extracted breath-to-breath peak expired carbon dioxide values. The resulting signals were visually inspected for artefacts or noise and corrected by cubic spline interpolation and downsampled to 10 Hz and subsequently ﬁltered with a dual-pass, 4th order digital Butterworth ﬁlter with a 2 Hz cut-off frequency. Data from each trial were aligned to stimulus onset (eyes open), and then averaged to generate one response per subject for both the PCA velocity and MCA velocity. From these averaged responses the following dependent variables were measured: (i) area under the curve to 30 s (AUC_30_); (ii) baseline CBv; (iii) initial slope of the CBv response; (iv) peak CBv (v) % increase in CBv; and (vi) time to peak CBv (35, 44).

### Statistical analyses

Paired-sample *t*-tests were initially performed to determine potential differences in each dependent variable for groups of participants who scored low on the BISA (0–4) vs. high on the BISA (5–8) or did or did not experience strangulation. A stepwise multiple linear regression analysis was subsequently completed to determine the impact of the psychopathological factors (e.g., PTSD, anxiety, and depression) on the relationship between the dependent variables and the experiences of IPV-BI as assessed with the BISA. All statistical tests were performed using SPSS, version 27 (IBC Corporation, Armonk, New York, USA). Data are presented as mean ± standard deviation, with *a priori* significance set at *p* = 0.05.

## Results

Table 1 outlines the demographic and clinical information of the participants. Participants were 37.42 ± 8.49 years old and had 13.36 ± 2.11 years of education. The majority (∼59%) were Caucasian, and a significant minority (∼27%) were Indigenous. Given ∼6% of the population in this region of Canada is Indigenous, this finding is consistent with the overrepresentation of Indigenous women experiencing IPV (47). Most of the participants (∼95%) scored above 20 on the WEB scale fulfilling the criteria for having experienced IPV. The average BISA score was 3.7 ± 2.17 with only 2 participants scoring 0 indicating that approximately 95% of participants experienced at least one episode of IPV resulting in signs and symptoms consistent with BI. The average time since the most recent IPV-BI episode was 18.39 ± 22.49 months with a very broad range (4–117 months) indicating all of the episodes were remote in time and none of the participants were in the acute/subacute stage after a potential BI. Finally, as a group, the participants had been engaged in substance use for 15.97 ± 9.84 years with the majority of them (∼73%) limiting this use to alcohol and/or tobacco. With respect to psychopathology, consistent with previous work (12, 19–23), most of the participants (∼93%) had elevated levels of PTSD and ∼49% had moderate or severe levels of depression and anxiety. Finally, in addition to a history of IPV-BI, many of the participants (∼62%) also had a history of one or more brain injuries from other causes.

**Figure 1:**
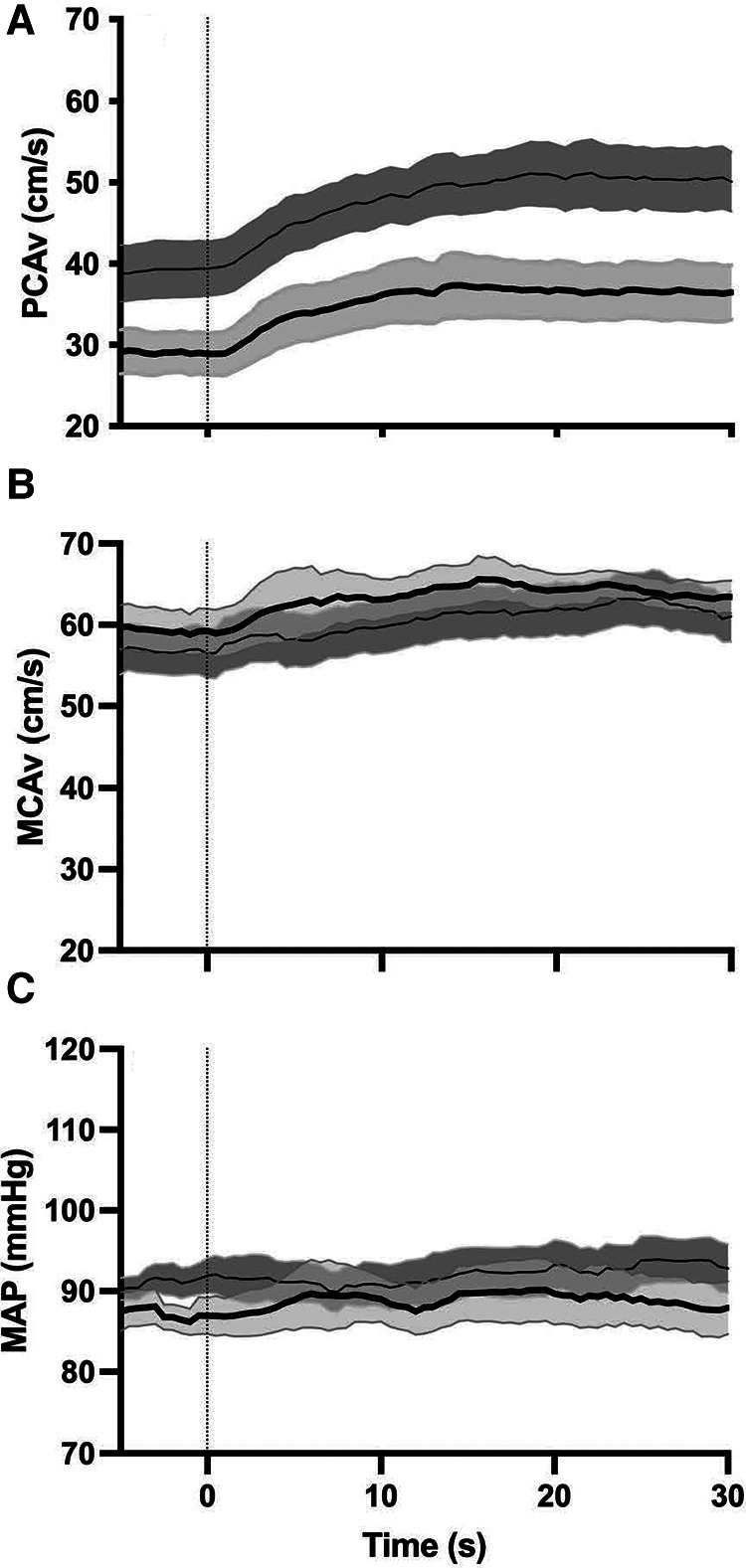
Group averages (*N* = 37 female IPV survivors) for NVC response in the PCA (**A**) and MCA (**B**) and corresponding MAP response (**C**) thin solid line and dark grey shaded region: average NVC response and intrasubject SEM for low BISA group. Thick solid line and light grey shaded region: average NVC response and intrasubject SEM for the high BISA group. Dashed vertical line: onset of opening eyes and visual search task.

Analysis of the NVC metrics demonstrated that they were normally distributed as determined by Shapiro–Wilk's test (*p* > 0.05). Initially, we were interested in whether there were any differences between participants who scored low on the BISA (0–4) (*n* = 22) and those who scored high on the BISA (5–8) (*n* = 15). Figure 1 shows the averaged NVC coupling responses in these two groups for the PCA velocity and MCA velocity and the associated time course of mean arterial pressure (MAP). The most obvious differences are in the NVC response in the PCA which shows a lower CBv at both baseline and peak for the high BISA group relative to the low BISA group. These differences were not apparent in the MCA. In addition, the magnitude of the response to opening the eyes and starting the visual search is substantially more muted overall in the MCA relative to the PCA consistent with previous work using this paragidm (35, 44). Finally, MAP did not vary during task performance relative to baseline indicating the changes in CBv were not affected by alterations in systemic blood pressure.

**Figure 2:**
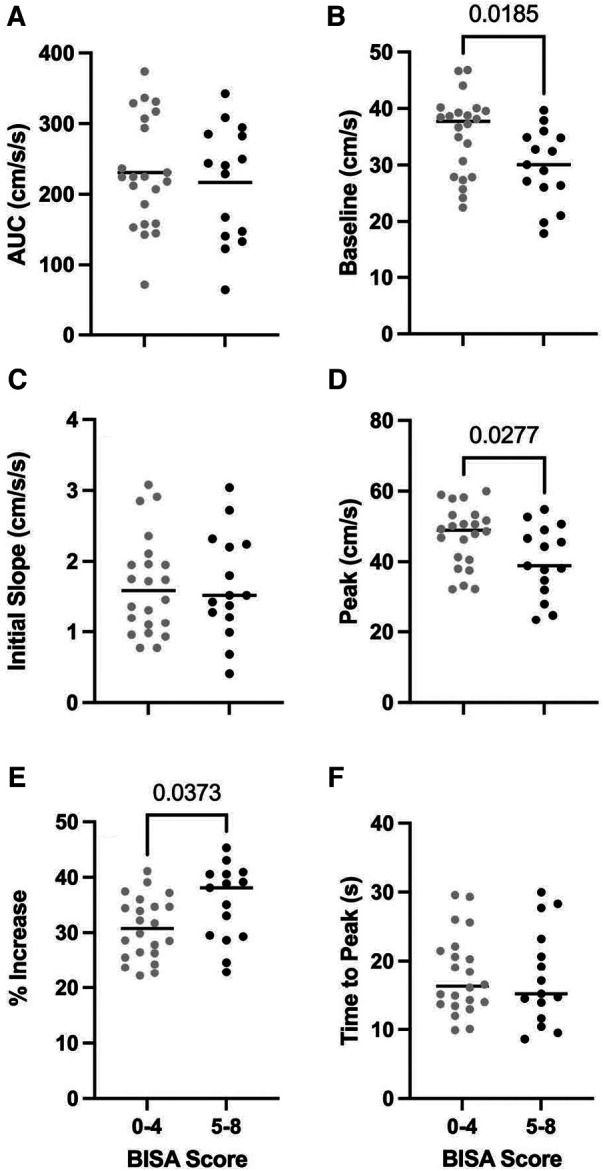
Group averages (*N* = 37 female IPV survivors) for PCA NVC metrics for AUC_30_ (**A**), baseline PCA velocity (**B**), initial slope (**C**), peak PCA velocity (**D**), % increase in PCA velocity (**E**), and time to peak PCA velocity (**F**) light grey circles: low BISA group. Black circles: high BISA group. Horizontal lines: median values for each group.

Figure 2 shows the NVC metrics for PCA velocity across the low and high BISA groups. Consistent with the observations from the average NVC responses in the PCA velocity shown in Figure 1, differences between the groups were observed for the baseline PCA velocity (*t*-test = 2.47, *p* = 0.0185) and the peak PCA velocity (*t*-test = 2.297, *p* = 0.0277). In addition, the % increase in PCA velocity was greater in the high BISA group than the low BISA group (*t*-test = 2.165, *p* = 0.0373). None of the other metrics were significantly different for PCA velcoity and there were no differences between the groups for the MCA velocity or for MAP.

**Figure 3:**
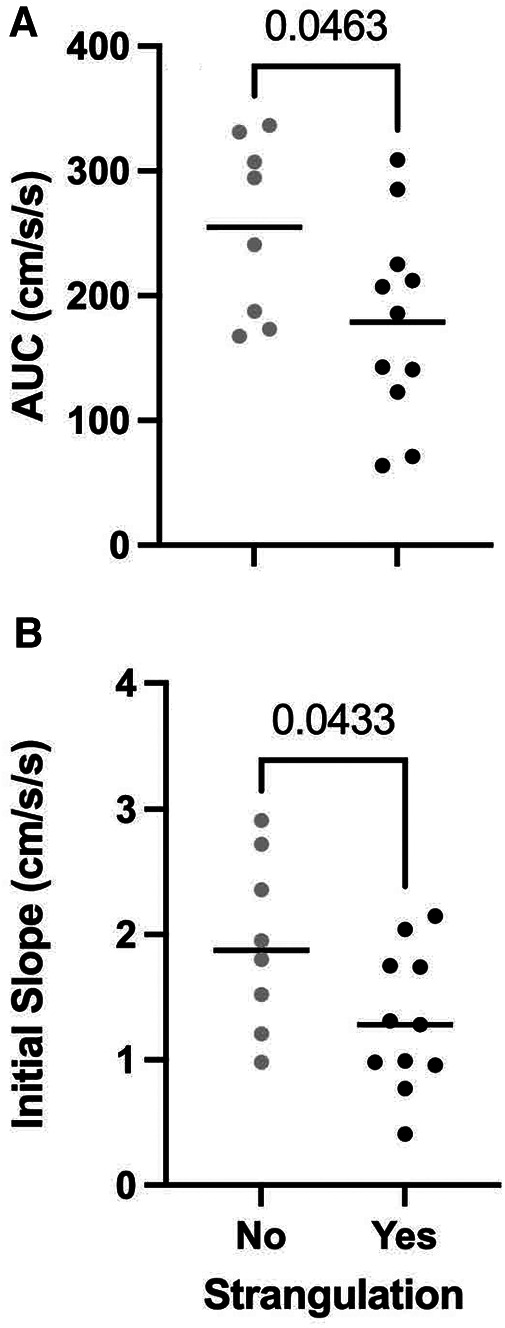
Group averages (*N* = 37 female IPV survivors) for PCA NVC metrics for AUC_30_ (**A**) and initial slope (**B**) for participants who did (black circles) or did not experience strangulation (light grey circles). Horizontal lines: median values for each group.

Next we asked what impact NFS had on the NVC metrics in the PCA. Given that NFS tends to be associated with elevated BISA scores,^1^ we restricted this analysis to a subset of participants who differed in whether they experienced NFS or not but overlapped in their scores on the BISA. The group that had not experienced NFS (*n* = 8) had an average BISA score of 4 ± 2.12 whereas the group that had experienced NFS (*n* = 11) had an average BISA score of 4.18 ± 3.54 (*t*-test = 0.382, *p* > 0.38). Comparison of these two groups on the NVC metrics demonstrated those who had experienced NFS had smaller AUC_30_ (*t*-test = 2.149, *p* = 0.0463) and initial slope (*t*-test = 2.184, *p* = 0.0433) values (Figure 3). None of the other NVC metrics differed between the two groups.

Finally, in an effort to better understand the contributions of comorbid psychopathological and demographic factors in modulating the relationship between the NVC metrics and BISA scores we completed stepwise multiple linear regression analyses using 3 models (i) in Model 1, PTSD scores were included as a covariate; (ii) in Model 2, depression and anxiety scores were included as covariates; and (iii) in Model 3, demographic variables (age, ethnicity, education, substance use and non IPV-related BI) were included as covariates. Because the only differences in the preliminary analysis were observed for the PCA and not the MCA, we restricted the regression analysis to NVC metrics from the PCA. Moreover, the analysis was limited to a subset of the participants (*n* = 24) for whom we had complete datasets for all the variables of interest. Table 2 shows the results of this analysis. For Model 1, the only significant effect was found for the % increase in PCA velocity which was associated with the BISA score. In particular, for every 1 unit change in the BISA score, the % increase in PCA velocity was increased by 1.65 units (*p* = 0.03) and this relationship was not modulated by the level of PTSD experienced by the participants. This result is consistent with the significant difference between the low and high BISA groups for this variable in the preliminary analysis (Figure 2E). By contrast, several significant effects were found for Model 2. In particular, although none of the NVC metrics were significantly affected by the BISA score in this model, AUC_30_, initial slope, and % increase in PCA velcoity were all modulated by the levels of depression, whereas the % increase in PCA velcoity was further modulated by the levels of anxiety. Finally, for Model 3 (not shown), no significant effects were observed.

**Table 2:** Stepwise multiple linear regression, models 1 and 2 for PCA (*N* = 24).

		*R* ^2^	*B*	*β*	Sig.	95% CI	
AUC (cm/s/s)							
Model 1	BISA	0.05	5.605	.160	.496	−11.227	22.436
	CAPS total		−.350	−.228	.335	−1.087	.387
Model 2	BISA	0.29	.076	.002	.991	−14.390	14.542
	BAI total		2.449	.432	.062	−.139	5.037
	BDI total		−4.148	−.568	.013*	−7.336	−.960
Baseline velocity (cm/s)							
Model 1	BISA	0.08	−.977	−.251	.281	−2.814	.860
	CAPS total		−.010	−.060	.793	−.091	.070
Model 2	BISA	0.14	−.938	−.241	.282	−2.706	.831
	BAI total		−.010	−.016	.946	−.327	.306
	BDI total		−.203	−.251	.289	−.593	.186
Initial Slope (cm/s/s)							
Model 1	BISA	0.09	.061	.193	.403	−.088	.210
	CAPS total		−.004	−.321	.170	−.011	.002
Model 2	BISA	0.32	.022	.068	.729	−.107	.151
	BAI total		.012	.227	.305	−.011	.035
	BDI total		−.041	−.619	.007*	−.069	−.013
Peak velocity (cm/s)							
Model 1	BISA	0.04	−.725	−.143	.545	−3.174	1.724
	CAPS total		−.019	−.086	.716	−.126	.088
Model 2	BISA	0.13	−.811	−.160	.474	−3.133	1.510
	BAI total		.059	.072	.770	−.356	.474
	BDI total		−.359	−.340	.159	−.870	.153
% Increase in velocity							
Model 1	BISA	0.20	1.652	.481	.033*	.144	3.160
	CAPS total		−.015	−.098	.646	−.081	.051
Model 2	BISA	0.42	1.164	.339	.074	−.125	2.452
	BAI total		.271	.487	.024*	.040	.501
	BDI total		−.295	−.413	.042*	−.579	−.011
Time to peak (s)							
Model 1	BISA	0.07	−.353	−.129	.578	−1.652	.946
	CAPS total		.034	.287	.222	−.022	.091
Model 2	BISA	0.06	−.239	−.087	.706	−1.540	1.063
	BAI total		.079	.179	.485	−.153	.312
	BDI total		.070	.122	.617	−.217	.357

Model, “Stepwise” method in SPSS Statistics; *B*, unstandardized regression coefficient; *β*, standardized regression coefficient; CI, confidence interval; LL, lower limit; UL, upper limit; *R*^2^, coefficient of determination.

* *p* < 0.05.

## Discussion

This study provides the first examination of NVC characteristics in women who have experienced IPV-BI. We demonstrated (i) the NVC response is muted in women who have had more exposure to IPV-BI; (ii) experiencing NFS modulated the NVC response above and beyond that observed due to head impacts alone; and (iii) these effects were associated with the levels of comorbid depression and anxiety. The fact this aspect of cerebrovascular function is affected by the combination of the head impacts and NFS occurring during IPV as well as the psychological distress associated with this experience is consistent with previous work in this population (12, 13, 17, 25, 48) and reflects the complex interaction between the physical injuries and the mental health consequences resulting from episodes of IPV.

Previous work has demonstrated BI from other injury mechanisms (e.g., sport-related concussion, accidents, military) is associated with disruptions to the NVC response. Our group has shown that NVC metrics are elevated for up to two weeks following sport-related concussion compared to non-injured controls (35), as well as following a controlled bout of soccer heading (49), but not after a season of subconcussive head impacts in collision sport athletes (50), with this difference likely due to the potential protective effects associate with physical activity (51, 52). Interestingly, and more directly relevant to the current study, Roby and colleagues (53) reported that military personnel with a history of 3 or more previous BIs displayed a reduced NVC response compared to those with 1–2 previous BIs. Analogous results have recently been demonstrated using functional near infrared spectroscopy in retired collision sport athletes with a history of BI (54). Thus, the acute/subacute increase in the NVC response, likely linked to aspects of the neurometabolic cascade following BI (55), evolves over time into a muted NVC response in those who have been exposed to multiple BI events including, as shown by the results from the current study, women who have survived IPV-BI. Because of the nature of the Where's Waldo search task, we did not formally assess task performance. Indeed, most participants were unsuccessful in locating Waldo during the time spent searching. As a result, we were unable to determine if the reduced NVC response in those women with greater exposure to IPV-BI was associated with reduced task performance. Although the present study did not allow us to mechanistically examine the implications of this reduced NVC response, it suggests those women with greater exposure to IPV-BI would require a larger delivery of oxygen and nutrients to accomplish a task with the same degree of success as women with less exposure to IPV-BI (31, 56). Interestingly, this may explain why BI symptoms such as fatigue, low energy, drowsiness, and difficulty concentrating are amongst the most common and highly correlated with the BISA in women experiencing IPV-BI (17). Future studies should be designed to address the links between changes in NVC responses and task performance in participants who have experienced IPV-BI by using behavioural protocols in which response accuracy and timing can be measured.

Research directly examining cerebrovascular function in women who have experienced IPV is remarkably limited. However, there are studies which have indirectly shed light on this potential link. For example, it has been shown IPV leads to an increased risk of hypertension (57) and this relationship is exacerbated in those who have also suffered a BI (58). Moreover, comorbid mental health factors associated with IPV, including PTSD, depression, and anxiety, are also known to be associated with hypertension (59). Finally, hypertension has been linked to alterations in NVC (32, 33, 60). Thus, given these intersecting set of factors, it is perhaps not surprising we found the NVC response was disrupted in IPV-BI in the present study.

Finally, because IPV tends to be a cyclical/repetitive process, there is growing concern around the potential cumulative impact of repeated BIs or subconcussive blows to the head, face, and neck on longer term neurodegenerative disease processes including chronic traumatic encephalopathy (CTE) and AD and related dementias. Although there are case reports of putative CTE in survivors of IPV-BI (27, 61), a recent more in depth case series found consistent neuropathological evidence of white matter disruption consistent with BI as well as vascular damage but, importantly, no evidence meeting the criteria for CTE (62). Moreover, the women whose brains were included in this case series had complex histories of comorbid factors including substance use, epilepsy, cerebrovascular, and psychiatric conditions reinforcing the need to take such factors into account in research in this population. With respect to IPV-BI being a risk factor for AD, the evidence is still very much in its infancy. One study examined this association and found women who had a self/family-reported history of head trauma from IPV were more likely to have AD than those without such a history. Previous work has demonstrated that BI from other injury mechanisms is a known risk factor for AD (63). Importantly with respect to the findings from the current study, NVC responses have been shown to be altered in hypertension and AD (32–34). A potential mechanism for the altered NVC responses reported in these populations could be that associated with disruptions to the glymphatic system as it plays a vital role in the removal of waste/by-products from the NVC response and maintenance of the white matter within the brain (64). Furthermore, the links between NVC and removal of metabolic waste by the glymphatic system (65) have been shown to be disrupted in neurodegenerative pathology (66, 67) and are likely contributing to the challenges facing survivors of IPV (68, 69). Finally, there is evidence that BI-induced cerebrovascular dysregulation contributes to the cognitive impairments associated with AD (28, 29). Taken together, the results from the current study, when considered in the context of this previous work suggests that IPV-BI may increase the risk of developing AD, however, a more definitive answer awaits further more in depth epidemiology and neuropathological work. Over the longer term, the links between altered NVC responses and exposure to IPV-BI highlight the potential for pharmacological and non-pharmacological treatment targets designed to improve cerebral blood flow responses. Although such treatments are still under development, some preliminary evidence suggests they may be effective following BI from other injury mechanisms (reviewed in 30) and, therefore, may be useful in the context of IPV-BI.

### Limitations

The current study does have its limitations. First, the sample size is relatively small and, thus, we may lack the power needed to detect subtle but potentially important effects. Second, recruitment was limited to several key community organizations serving women who have experienced IPV. Because of this, the extent to which we can generalize our findings to the broader population experiencing IPV is limited. Third, there was wide variation in the time since the most recent IPV-BI episode potentially introducing substantial variability in our measures of NVC responses. Fourth, because we used a cross-sectional approach and the participants had numerous comorbid factors, we can not draw causal links between IPV-BI and the observed effects on NVC.

## Conclusions

This study is the first to our knowledge to characterize a component of cerebrovascular function in women who have experienced IPV-BI. We demonstrated several important NVC metrics were affected by the extent of previous exposure to IPV-BI and associated NFS and these effects were modulated by the levels of depression and anxiety. Taken together, these results show cerebrovascular function is affected by a history of BI resulting from IPV. This indicates there are functional changes in the neurons of IPV survivors following BI, which reduces the efficiency of their brain metabolism during task performance. This key finding suggests this may place women who survive this experience at increased risk of developing longer-term neurodegenerative disorders.

## Data Availability

The raw data supporting the conclusions of this article will be made available by the authors, without undue reservation.

## Footnotes

^1^Adhikari SP, Maldonado-Rodriguez N, Daugherty JC, Molinares NQ, Wallace C, Smirl J, et al. A four country study of strangulation-related alterations in consciousness in women who have experienced intimate partner violence: co-occurrence with traumatic brain injuries and measures of psychological distress. (2023).
